# Ethanolic Extract of Folium Sennae Mediates the Glucose Uptake of L6 Cells by GLUT4 and Ca^2+^

**DOI:** 10.3390/molecules23112934

**Published:** 2018-11-09

**Authors:** Ping Zhao, Qian Ming, Junying Qiu, Di Tian, Jia Liu, Jinhua Shen, Qing-Hua Liu, Xinzhou Yang

**Affiliations:** 1 Institute for Medical Biology & Hubei Provincial Key Laboratory for Protection and Application of Special Plants in the Wuling Area of China, College of Life Sciences, South-Central University for Nationalities, Wuhan 430074, China; 18062196191@163.com (Q.M.); qiu927633@163.com (J.Q.); tiandi_2983@126.com (D.T.); 13512566172@163.com (J.L.); shenjinhua2013@163.com (J.S.); qinghualiu95@163.com (Q.-H.L.); 2 National Demonstration Center for Experimental Ethnopharmacology Education, South-Central University for Nationalities, Wuhan 430074, China; 3 School of Pharmaceutical Sciences, South-Central University for Nationalities, 182 Min-Zu Road, Wuhan 430074, China

**Keywords:** FSE, T2DM, GLUT4, Ca^2+^, L6 cell

## Abstract

In today’s world, diabetes mellitus (DM) is on the rise, especially type 2 diabetes mellitus (T2DM), which is characterized by insulin resistance. T2DM has high morbidity, and therapies with natural products have attracted much attention in the recent past. In this paper, we aimed to study the hypoglycemic effect and the mechanism of an ethanolic extract of Folium Sennae (FSE) on L6 cells. The glucose uptake of L6 cells was investigated using a glucose assay kit. We studied glucose transporter 4 (GLUT4) expression and AMP-activated protein kinase (AMPK), protein kinase B (PKB/Akt), and protein kinase C (PKC) phosphorylation levels using western blot analysis. GLUT4 trafficking and intracellular Ca^2+^ levels were monitored by laser confocal microscopy in L6 cells stably expressing IRAP-mOrange. GLUT4 fusion with plasma membrane (PM) was observed by myc-GLUT4-mOrange. FSE stimulated glucose uptake; GLUT4 expression and translocation; PM fusion; intracellular Ca^2+^ elevation; and the phosphorylation of AMPK, Akt, and PKC in L6 cells. GLUT4 translocation was weakened by the AMPK inhibitor compound C, PI3K inhibitor Wortmannin, PKC inhibitor Gö6983, G protein inhibitor PTX/Gallein, and PLC inhibitor U73122. Similarly, in addition to PTX/Gallein and U73122, the IP3R inhibitor 2-APB and a 0 mM Ca^2+^-EGTA solution partially inhibited the elevation of intracellular Ca^2+^ levels. BAPTA-AM had a significant inhibitory effect on FSE-mediated GLUT4 activities. In summary, FSE regulates GLUT4 expression and translocation by activating the AMPK, PI3K/Akt, and G protein–PLC–PKC pathways. FSE causes increasing Ca^2+^ concentration to complete the fusion of GLUT4 vesicles with PM, allowing glucose uptake. Therefore, FSE may be a potential drug for improving T2DM.

## 1. Introduction

Diabetes mellitus (DM) is a chronic metabolic disorder resulting from insufficient insulin secretion or insulin dysfunction. It may lead to a series of complications such as renal failure, cardiovascular disease, blindness, hypertension, non-alcoholic fatty liver disease (NAFLD), and obesity [1,2]. The global prevalence of DM is on the rise, especially in developing countries [3]. T2DM is the most prevalent form of DM and accounts for more than 90% of the cases of DM [4]. Insulin resistance is the typical feature of T2DM, which causes cells to stop responding adequately to the standard role of insulin. While the body continues to produce insulin, the cells in the body become resistant to its effects. Therefore, cells cannot effectively process insulin, resulting in hyperglycemia [5,6,7].

Blood glucose influx into cells requires glucose transporter family proteins (GLUTs) [1]. With the onset of hyperglycemia, the glucose uptake in the adipose tissue and muscles is mostly mediated by GLUT4, an isoform of a family of sugar transporter proteins (encoded on the SLC2A4 gene) containing 12 transmembrane domains. GLUT4 continuously recycles between intracellular vessels and the plasma membrane (PM) [8,9]. After insulin stimulation, GLUT4 proteins are mobilized to PM immediately, which enhances the rate of exocytosis and their fusion with PM (this process is synonymous with GLUT4 translocation). The increase in PM GLUT4 leads to glucose uptake [10]. Previous studies have reported that defective GLUT4 translocation is a feature of insulin resistance and an essential precursor of T2DM [4,7,11]. Thus, GLUT4 as a key regulatory target for glucose homeostasis is widely used in antidiabetic drug research.

Intracellular cytosolic free Ca^2+^ comes from both extracellular Ca^2+^ influx and Ca^2+^ release from intracellular stores (including sarcoplasmic reticulum, lysosomes, and mitochondria). As an important second messenger, it participates in many physiological activities of cells. Numerous studies have highlighted the role of cytosolic Ca^2+^ in GLUT4 synthesis, GLUT4 traffic (endocytosis and exocytosis), and glucose uptake. Wright reported that the treatment of L6 muscle cells with agents that increase Ca^2+^ leads to an increase in the GLUT4 protein content [12]. Li et al. found that Ca^2+^ signals promote GLUT4 exocytosis and reduce its endocytosis in muscle cells [13]. Johanna et al. described the role of Ca^2+^ influx for insulin-mediated glucose uptake in skeletal muscles [14]. Contreras-Ferrat et al. clearly showed that an inositol 1,4,5-triphosphate (IP3)-dependent Ca^2+^ release pathway is required for insulin-stimulated GLUT4 translocation and glucose uptake in cardiomyocytes [15]. Similarly, it has been reported that the AMPK, PKC, and insulin-dependent pathways were relatively independent of Ca^2+^-regulated GLUT4 traffic [16,17,18,19]. Furthermore, increases in intracellular Ca^2+^ levels, even at concentrations too low to induce contractions, provide the signal to activate GLUT4 translocation in skeletal muscles [17].

Because of serious economic burdens and side effects of chemical agent-based DM treatment strategies [7,20,21], natural drug products are gaining popularity because of their various advantages, such as fewer side effects, better patient tolerance, relatively lower cost, and acceptance due to the long history of use. An important cause of these products’ efficacy is that, unlike a single chemical entity aimed at a specific single target, many Chinese herbal medicines (such as the multi-flavonoid-rich plant extracts) are thought to alleviate the disorder of diabetes mellitus through an integrated effect upon multitarget sites [22,23]. Current studies have shown that phytochemicals, polysaccharides, flavonoids, terpenoids, tannins, steroids, and other chemicals naturally found in plants possess antidiabetic activity [8,24,25]. The two main antidiabetic agents represented by metformin and flavonoids were derived from medicinal plants. Folium Sennae, also called the senna leaf, is derived from the dried leaflets of *Cassia angustifolia Vahl* or *Cassia acutifolia Delile*, and belongs to the dicotyledonous leguminous family. It is native to India and Egypt, and is widely distributed in the Taiwan, Guangdong, Guangxi, and Yunnan provinces of China [26]. The Chinese Pharmacopoeia (2010) and Chinese Materia Medica state that senna leaves contain four sennosides (A, B, C, and D), rhein, emodin, chrysophanol, aloe-emodin, physcion, tinnevellin glucoside, kaempferol, phytosterol and its glycosides, pine camphor, salicylic acid, and several other ingredients. In addition to being well known as a natural laxative [27], the senna leaf has been found to exhibit antioxidant [28], antibacterial [29], anti-inflammatory [30], antitumor [31], analgesic [32], antimalarial [33], and antidiabetic [34] activities. Ayinla et al. reported that the ethanolic leaf extract of Senna fistula improved hematologic parameters, lipid profiles, and oxidative stress in alloxan-induced diabetic rats [35]. Thilagam et al. discovered that the ethanolic leaf extract of Senna surattensis inhibited the carbohydrate digestive enzymes and increased the peripheral glucose uptake in the isolated rat hemidiaphragm model [34]. Malematja et al. showed that Senna italica leaf acetone extract promoted glucose uptake and anti-obesity through the PI3K-dependent pathway [36]. However, only a few studies have assessed the possible hypoglycemic properties and hypoglycemic mechanisms of Folium Sennae (FSE).

In the present study, we observed that FSE displayed a strong effect in promoting glucose uptake, GLUT4 expression and translocation, and cytosolic Ca^2+^ levels in L6 rat skeletal muscle cells. We observed that FSE induced GLUT4 expression and translocation through the AMPK, PI3K/Akt, and PKC signaling pathways. FSE also increased cytosolic Ca^2+^ concentration by extracellular Ca^2+^ influx or/and intracellular Ca^2+^ release of G protein-IP3-IP3R signals, which assisted GLUT4 movement and stimulated glucose uptake. We elucidated the mechanism of action and validated the beneficial effects of FSE as an antidiabetic agent.

## 2. Results

### 2.1. FSE Increases GLUT4 Expression Levels and Glucose Uptake in L6 Cells

**Figure 1:**
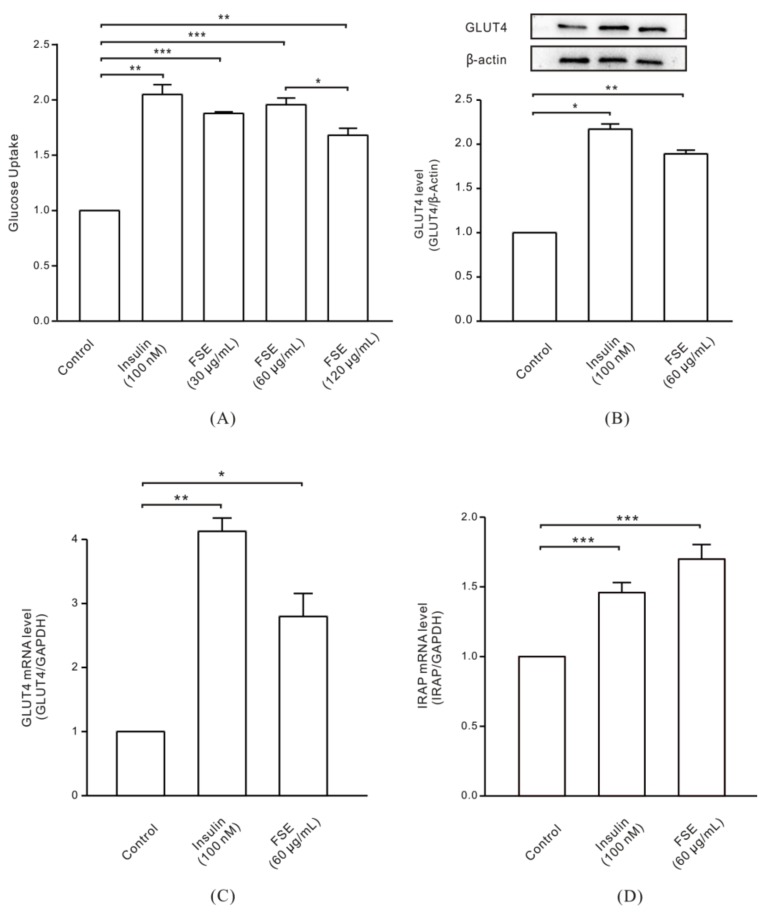
Enhancing the effects of Folium Sennae (FSE) on glucose uptake and GLUT4 expression in L6 cells. Insulin as a positive control. (**A**) Cells were incubated with 100 nM insulin or 30, 60, and 120 μg/mL FSE for 1 h, and glucose uptake was measured using a glucose assay kit. (**B**) Cells were stimulated with 100 nM insulin or 60 μg/mL FSE for 30 min. Whole cell lysates were subjected to western blot analysis for GLUT4, and the protein expression level was normalized against β-actin. (**C**) Cells were treated with 100 nM insulin or 60 μg/mL FSE for 30 min. GAPDH was used to normalize the mRNA level, and the relative expression of GLUT4 mRNA was investigated by real-time PCR. (**D**) Cells were treated with 100 nM insulin or 60 μg/mL FSE for 30 min. GAPDH was used to normalize the mRNA level, and the relative expression of IRAP mRNA was investigated by real-time PCR. The data were obtained from three independent repeated experiments. Significance analysis: * *p* < 0.05; ** *p* < 0.01; *** *p* < 0.001.

To confirm the possible hypoglycemic activity of FSE, we first studied its glucose uptake effect. As shown in Figure 1A, L6 cells were serum-deprived and then incubated with 100 nM insulin or different concentrations of FSE for 1 h. When compared with the control group, insulin (positive control) and 30, 60, and 120 μg/mL of FSE significantly promoted the glucose uptake of cells by 2.04-fold, 1.87-fold, 1.95-fold, and 1.68-fold, respectively. This result was based on the corresponding MTT assay after drug treatment. MTT results showed that neither FSE nor insulin caused toxicity to L6 cells (Figure S1). In the next step, we chose 60 μg/mL as the best concentration of FSE. To understand whether FSE affected the expression of the glucose regulator GLUT4, the total protein and mRNA of GLUT4 and the mRNA of IRAP in L6 cells were extracted after stimulation with insulin or FSE. The results showed that 100 nM insulin and 60 μg/mL FSE increased the protein expression of GLUT4 by 2.13-fold and 1.89-fold, respectively (Figure 1B). The fold increases of GLUT4 mRNA levels were even more prominent, at 4.13-fold with insulin and 2.8-fold with FSE (Figure 1C). In addition, as a resident protein of GLUT4 storage vesicles, insulin-regulated aminopeptidase (IRAP) also changed at the mRNA level, with 1.46-fold from insulin treatment and 1.70-fold from FSE treatment (Figure 1D).

### 2.2. FSE Stimulates GLUT4 Translocation and Increases Intracellular Ca^2+^ Levels

**Figure 2:**
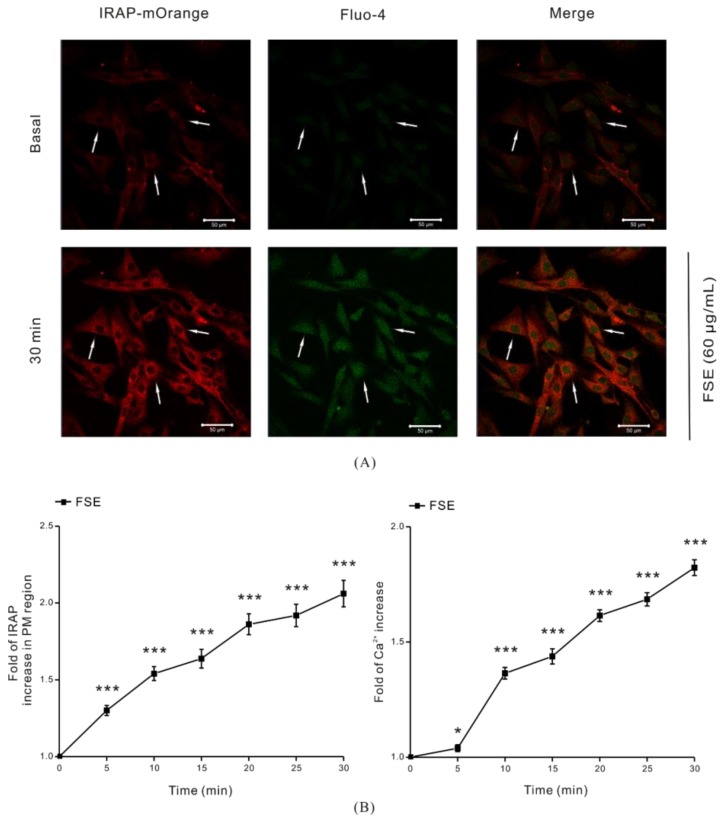
Stimulating effects of FSE on GLUT4 translocation and intracellular Ca^2+^ level. The red fluorescence of IRAP-mOrange stably expressed in L6 cells and the green fluorescence of Ca^2+^ were simultaneously observed by confocal microscope. Scale bar = 50 μm. (**A**) Intracellular Ca^2+^ was stained with Flou-4 AM for 20 min, followed by stimulation with 60 μg/mL FSE for 30 min. IRAP-mOrange fluorescence intensity and intracellular Ca^2+^ fluorescence concentration were detected at excitation wavelengths of 555 nm and 488 nm, respectively, and fluorescence superposition displayed specific positioning. (**B**) The cell images were recorded over 30 min, and the red fluorescence from the outside edges of cells and the green fluorescence of the whole cells were collected. Fluorescence quantization was done with Zeiss 2010 software. Significance analysis: * *p* < 0.05; *** *p* < 0.001.

Since intracellular GLUT4 translocation to the cell surface can exert glucose uptake function, we further analyzed GLUT4 translocation in L6 cells under FSE treatment. L6 cells stably expressing IRAP-mOrange (L6-mOrange-IRAP) were transfected with red fluorescent protein (mOrange)-tagged IRAP. IRAP was initially found in specialized vesicles containing GLUT4, which immediately migrated to the cell surface along with GLUT4 after receiving insulin [37]. Some evidences proved that IRAP was highly co-localized with GLUT4 [38,39]. We used Fluo-4 AM fluorescent dyes during loading of cells with Ca^2+^ and monitored the translocation of GLUT4 and intracellular Ca^2+^ changes in live cells by real-time fluorescence microscopy. As a comparative insulin treatment, the image showed that the intracellular IRAP-mOrange signal was enhanced and signal accumulation appeared in adjacent PM region. Green fluorescence was significantly brightened after 100 nM insulin treatment in intracellular Ca^2+^ detection (Figure S2). Similarly, the IRAP fluorescence intensity in cytoplasm was obviously raised after the addition of 60 μg/mL FSE, and a substantial amount of red fluorescence accumulated at the cell periphery as revealed by IRAP-mOrange signals. Meanwhile, the green fluorescence of Ca^2+^ was densely distributed in the cells (Figure 2A). The fold growth curve increased with IRAP level at the PM region or with intracellular Ca^2+^, and it increased in a time-dependent manner (Figure 2B). Our studies suggested that FSE promoted glucose uptake not only by stimulating GLUT4 expression and translocation but also by increasing intracellular Ca^2+^ levels.

### 2.3. The Role of Cytosolic Ca^2+^ in FSE-Mediated GLUT4 Translocation

**Figure 3:**
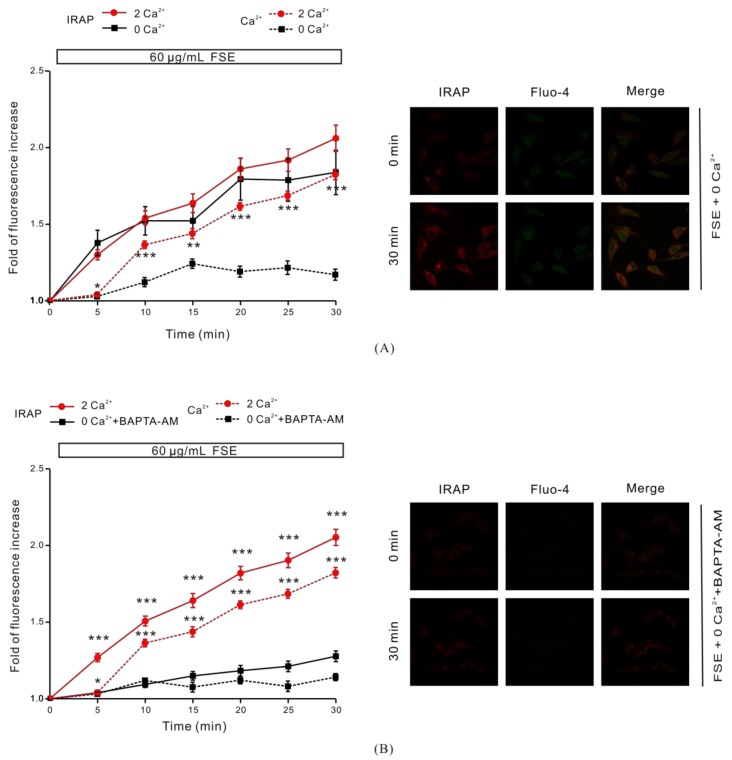
Role of intracellular Ca^2+^ on FSE-induced GLUT4 translocation. (**A**) After intracellular Ca^2+^ was loaded with Fluo-4 AM, cells were treated with 60 μg/mL FSE for 30 min under 0 mM extracellular Ca^2+^ conditions. * *p* < 0.05; ** *p* < 0.01; *** *p* < 0.001. (**B**) Cells were incubated for 30 min under the condition of 0 mM extracellular Ca^2+^ + 10 μM BAPTA-AM chelated intracellular Ca^2+^, followed by stimulation with 60 μg/mL FSE for 30 min to quantify IRAP-mOrange fluorescence in the PM region and intracellular Ca^2+^ levels. Significance analysis: * *p* < 0.05; ** *p* < 0.01; *** *p* < 0.001.

In order to determine whether the increase of intracellular Ca^2+^ concentration after FSE stimulation was related to GLUT4 translocation, we blocked the different sources of intracellular Ca^2+^ before treatment with 60 μg/mL FSE to observe the GLUT4 translocation. FSE-induced increase of intracellular Ca^2+^ was partially inhibited with the removal of extracellular Ca^2+^, but the FSE-mediated increase of IRAP fluorescence in the PM region remained unchanged (Figure 3A). This phenomenon can be explained by the observation that for FSE to evoke the rise of intracellular Ca^2+^, it needs at least to mobilize extracellular Ca^2+^ influx. In addition, when 0 mM extracellular Ca^2+^+BAPTA-AM was used to chelate cytosolic Ca^2+^, the FSE-induced increase of intracellular Ca^2+^ was completely inhibited, and the increase of IRAP fluorescence in the PM region was also obviously blocked (Figure 3B). These findings supported the idea that cytosolic Ca^2+^ plays an important role in the process of FSE-induced GLUT4 translocation to the PM.

### 2.4. FSE Enhances GLUT4 Translocation and Expression through the AMPK, PI3K/Akt, and PKC Pathways

**Figure 4:**
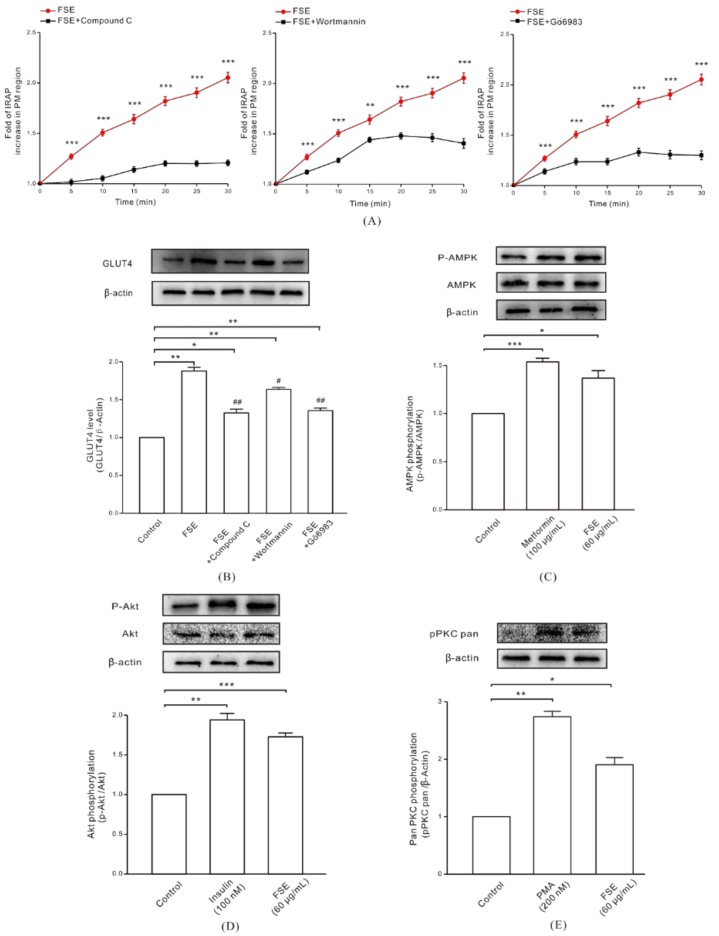
Effect of FSE on the AMPK, PI3K/Akt, and PKC signaling pathways and related proteins. (**A**) Cells were incubated with 10 μM compound C (AMPK inhibitor), 100 nM Wortmannin (PI3K inhibitor), or 10 μM Gö6983 (PKC inhibitor) for 30 min, and then treated with 60 μg/mL FSE. We calculated the fold of IRAP fluorescence increases in the PM region. * *p* < 0.05; ** *p* < 0.01; *** *p* < 0.001. (**B**) Cells were incubated with 10 μM compound C, 100 nM Wortmannin, or 10 μM Gö6983 inhibitor for 30 min and then were stimulated with 60 μg/mL FSE for 30 min. Whole cell lysates were subjected to western blot analysis for GLUT4 protein expression levels. * *p* < 0.05; ** *p* < 0.01, vs control group; ^#^ *p* < 0.05; ^##^ *p* < 0.01, vs. FSE group. (**C**) L6 cells were treated with 100 μg/mL metformin (overnight) or 60 μg/mL FSE (30 min) and then analyzed for phosphorylated-AMPK, total AMPK, and β-actin protein level by western blot analysis. * *p* < 0.05; *** *p* < 0.001. (**D**) Cells were treated with 100 nM insulin or 60 μg/mL FSE for 30 min, followed by western blot analysis of phosphorylated Akt, total Akt, and β-actin. ** *p* < 0.01; *** *p* < 0.001. (**E**) Cells were treated with 200 nM PMA (4 h) or 60 μg/mL FSE (30 min), and western blot was used to analyze the phosphorylated protein level of PKC in L6 cells. Data were from three independent repeated experiments. * *p* < 0.05; ** *p* < 0.01.

Next, we attempted to shed light on some of the signaling pathways involved in GLUT4 translocation and expression. After treatment with AMPK inhibitor Compound C (10 μM, 30 min), PI3K inhibitor Wortmannin (100 nM, 30 min), or PKC inhibitor Gö6983 (10 μM, 30 min), the increase of IRAP fluorescence in the PM region induced by 60 μg/mL FSE was inhibited (Figure 4A), and the inhibition from compound C (Figure 4A, left) and Gö6983 (Figure 4A, right) was stronger than that from Wortmannin (Figure 4A, middle). This indicated that AMPK, PI3K/Akt, and PKC may be involved in FSE-mediated GLUT4 expression and translocation. Following this, we used western blot analysis to verify our conjecture. Compared with the control group, the expression levels of GLUT4 protein in L6 cells were increased by 1.87-fold (FSE), 1.33-fold (FSE+Compound C), 1.63-fold (FSE+Wortmannin), and 1.35-fold (FSE+Gö6983) after treatment with FSE and/or these different inhibitors (Figure 4B). Consistent with Figure 4, the three inhibitors also exhibited their inhibitory effects on FSE-mediated GLUT4 protein expression (vs. FSE). Western blotting of the signaling pathway-related proteins revealed that the phosphorylation levels of AMPK, Akt, and PKC in L6 cells after treatment with 60 μg/mL FSE were upregulated by 1.36-fold, 1.72-fold, and 1.92-fold, respectively. Also, FSE’s effects were slightly lower than 1.54-fold of what was seen with 100 μg/mL metformin, 1.94-fold of what was seen with 100 nM insulin, and 2.74-fold of what was seen with 200 nM phorbol ester (PMA) in the positive control groups (Figure 4C–E). The above results showed that the increase in FSE-induced GLUT4 expression was dependent on AMPK, Akt, and PKC activities, and the FSE-promoted GLUT4 translocation also occurred through the AMPK, PI3K/Akt, and PKC pathways.

### 2.5. G Protein and PLC Regulate FSE-Mediated Intracellular Ca^2+^ Increases and GLUT4 Translocation

**Figure 5:**
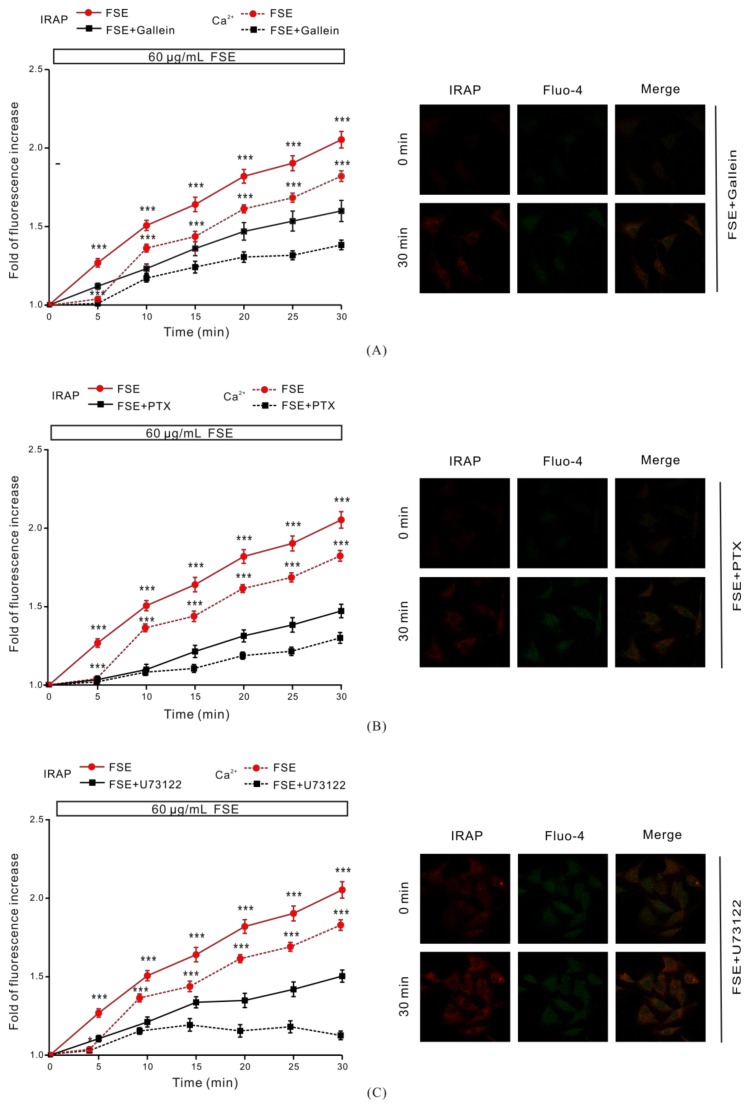
G protein-PLC signaling regulated FSE-mediated GLUT4 translocation and intracellular Ca^2+^ increase. (**A**) Cells were incubated for 6–8 h with 100 μM Gallein (Gβγ protein inhibitor) and then treated with 60 μg/mL FSE. * *p* < 0.05; *** *p* < 0.001. (**B**) Cells were incubated for 6–8 h with 100 μM PTX (Gα protein inhibitor), followed by the addition of 60 μg/mL FSE. *** *p* < 0.001. (**C**) Cells were treated with 2 μM U73122 (PLC inhibitor) for 30 min and were then stimulated by 60 μg/mL FSE. The IRAP-mOrange fluorescence in the PM region and the intracellular Ca^2+^ levels were quantified by Zeiss 2010 software. Significance analysis: * *p* < 0.05; *** *p* < 0.001.

G protein and PLC are upstream of the PKC pathway, and IP3R is one of the major receptors that trigger intracellular Ca^2+^ release. We investigated how G protein and PLC regulate FSE-induced Ca^2+^ increase and GLUT4 translocation. Cells treated with the Gβγ protein inhibitor 100 μM Gallein or Gα protein inhibitor 100 μM PTX for 6–8 h, or PLC inhibitor 2 μM U73122 for 30 min, significantly inhibited FSE-induced IRAP fluorescence intensity and intracellular Ca^2+^ elevation (Figure 5A–C). The results implied that FSE enhanced GLUT4 translocation via the G protein-PLC-PKC signaling pathway.

### 2.6. IP3R Is Involved in FSE-Triggered Intracellular Ca^2+^ Release

**Figure 6:**
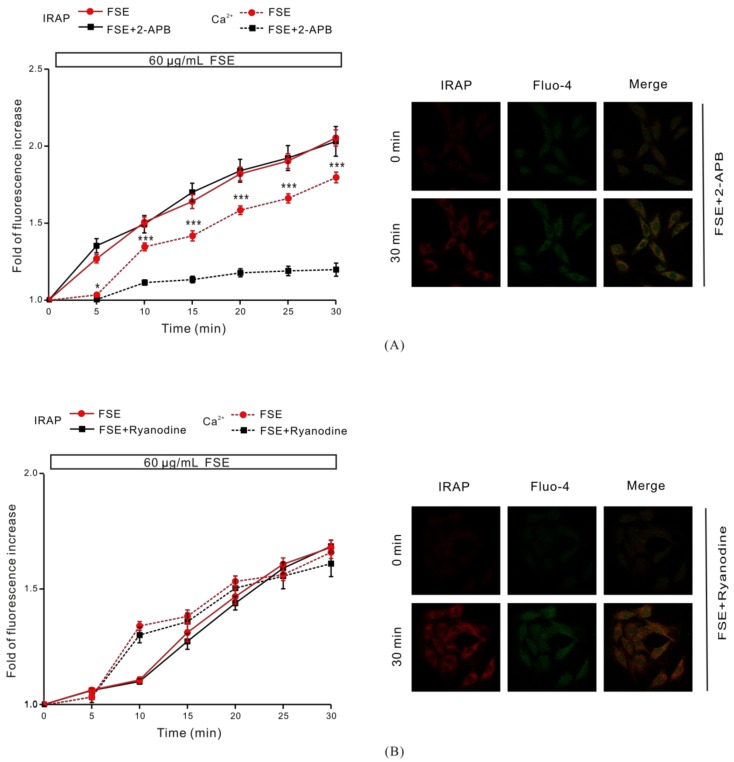
The IP3R receptor is involved in FSE-stimulated intracellular Ca^2+^ release. (**A**) Under extracellular 2 mM Ca^2+^, cells were treated with 100 μM 2-APB (IP3RS blocker) for 30 min and then treated with 60 μg/mL FSE. (**B**) Under extracellular 2 mM Ca^2+^, cells were incubated with 30 μM Ryanodine (RyR blocker) for 30 min, and the changes of IRAP-mOrange in the PM area and intracellular Ca^2+^ were measured after stimulation with 60 μg/mL FSE. Significance analysis: * *p* < 0.05; ** *p* < 0.01; *** *p* < 0.001.

The result shown in Figure 3 suggested the potential impact of intracellular Ca^2+^ release in the process of FSE-mediated GLUT4 translocation and the increase in intracellular Ca^2+^ levels. To support this hypothesis, we used 100 μM 2-APB to block IP3R-regulated intracellular Ca^2+^ release. We found that 2-APB had no effect on FSE-mediated GLUT4 translocation under 2 mM extracellular Ca^2+^, but it evidently inhibited FSE-triggered Ca^2+^ release (Figure 6A). The ryanodine receptor (RyR), another channel that releases Ca^2+^ in the sarcoplasmic reticulum (SR)/endoplasmic reticulum (ER) [40], has attracted much attention due to its manipulation of intracellular Ca^2+^ output. Inhibition of RyR with 30 μM ryanodine had no effect on either GLUT4 translocation or intracellular Ca^2+^ increase mediated by FSE (Figure 6B). These findings indicated that IP3R, rather than RyR, was involved in the FSE-triggered increases of intracellular Ca^2+^.

### 2.7. Ca^2+^ Is Required for GLUT4 Insertion into the PM

**Figure 7:**
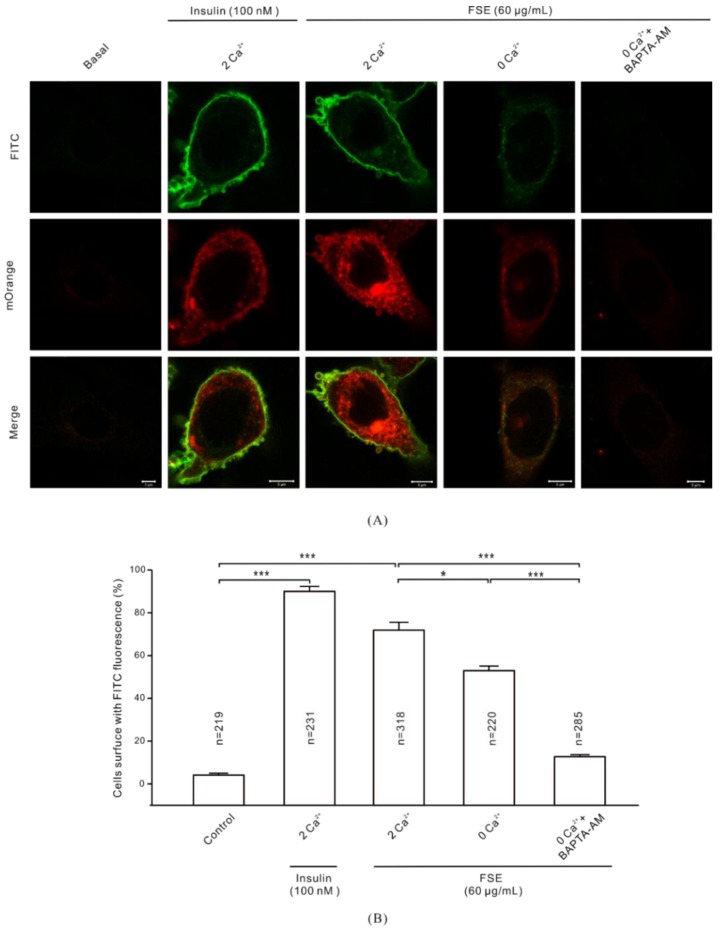
Involvement of Ca^2+^ in the fusion of GLUT4 and PM induced by FSE. (**A**) L6 cells transfected with plasmid GV348-myc-GLUT4-mOrange encoding an mOrange fusion protein with myc epitope-tagged GLUT4 (myc-GLUT4-FITC). Cells were stimulated with 100 nM insulin or 60 μg/mL FSE under 2 mM extracellular Ca^2+^, 0 mM Ca^2+^, and 0 mM Ca^2+^+BAPTA-AM conditions. Then, cells were fixed and subjected to specific immunofluorescence antibody staining. The mOrange red fluorescence and FITC green fluorescence were detected by confocal microscopy at 555 nm and 488 nm excitation wavelengths, respectively. Scale bar = 5 μm. (**B**) The percentage of FITC-positive cells in the total mOrange cell population was counted. The results shown were from three independent replicate experiments. Significance analysis: * *p* < 0.05; ** *p* < 0.01; *** *p* < 0.001.

Some studies have shown that Ca^2+^-assisted binding of GLUT4 to the PM is involved in preparation for glucose uptake by cells [41,42]. In our study, myc-labeled GLUT4 was used to observe the fusion of GLUT4 vesicles with the PM in L6 cells stably expressing myc-GLUT4-mOrange. Fluorescence imaging revealed that FITC labeling for anti-myc antibody was not detected on the cell surface in the absence of drug stimulation. When we treated the cells for 30 min with 100 nM insulin and 60 μg/mL FSE, we observed significant increases of FITC fluorescence signals on the cell surfaces (Figure 7A). However, the FSE-induced FITC fluorescence signal on the cell surface was seriously suppressed under the condition of 0 mM extracellular Ca^2+^/0 mM extracellular Ca^2+^ + 10 μM BAPTA-AM. On the other hand, the proportion of FITC-positive cells in the total number of mOrange cells showed (Figure 7B) that 0 mM Ca^2+^ + 10 μM BAPTA-AM had stronger inhibitory effects on FSE-mediated GLUT4 fusion with PM than the 0 mM Ca^2+^ group.

### 2.8. FSE-Induced Ca^2+^ Increases Improve Glucose Uptake in L6 Cells

**Figure 8:**
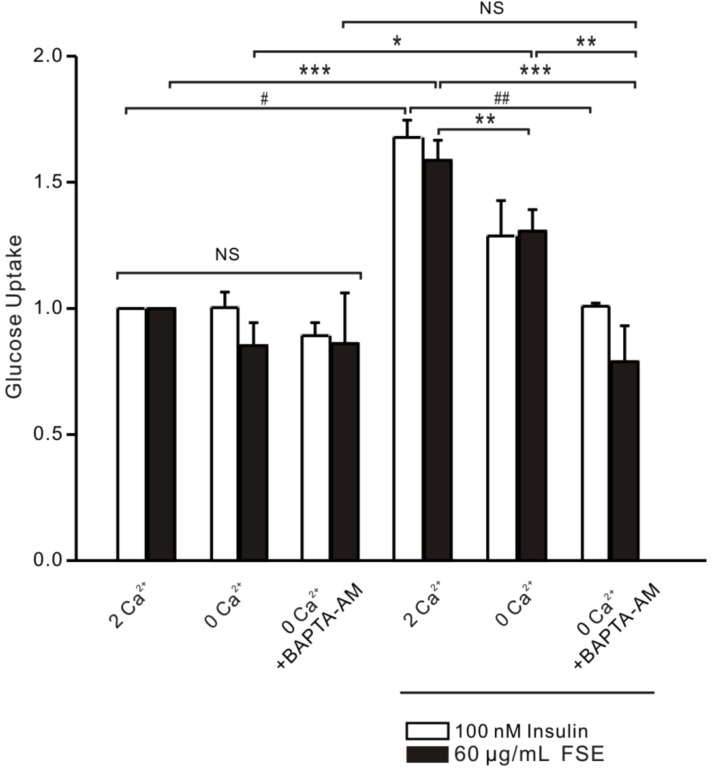
Increased effect of FSE on glucose uptake via Ca^2+^ signaling. Glucose uptake was measured using a glucose assay kit. L6 cells were serum-deprived for 2 h and incubated in 2 mM extracellular Ca^2+^, 0 mM extracellular Ca^2+^, or 0 mM extracellular Ca^2+^ + 10 μM BAPTA-AM chelated intracellular Ca^2+^ conditions. Cells were stimulated with vehicle, 60 μg/mL FSE, or 100 nM insulin for 1 h, and then the glucose uptake was measured. The experimental data are from six independent repeated experiments. Significant analysis: FSE group, * *p* < 0.05; ** *p* < 0.01; *** *p* < 0.001. Insulin group, ^#^ *p* < 0.05; ^##^ *p* < 0.01.

To clarify the relationship between FSE-mediated glucose uptake and Ca^2+^, a glucose uptake experiment was performed under various Ca^2+^ conditions. Results demonstrated that there was no significant difference in the glucose uptake between the groups in the absence of FSE or in the insulin-positive control group, whereas glucose uptake by cells was remarkably increased by 1.58-fold after the addition of FSE for 1 h in the culture medium with 2 mM extracellular Ca^2+^. Similarly, insulin treatment showed a 1.68-fold increase in glucose uptake. To a certain extent, 0 mM extracellular Ca^2+^ inhibited the effect of FSE by 1.3-fold or the effect of insulin by 1.28-fold. Meanwhile, insulin or FSE-mediated glucose uptake was completely blocked under 0 mM Ca^2+^+BAPTA-AM, which indicated that Ca^2+^ plays a crucial role in the FSE-induced glucose uptake process (Figure 8).

## 3. Discussion

Diabetes has long been a global health problem. In particular, the high proportion of T2DM characterized by insulin resistance in DM has become the focus of DM research [43]. Skeletal muscle is the primary tissue for insulin-stimulated glucose uptake in the body. It consumes glucose and is responsible for approximately 80% of the postprandial glucose intake and consumption, and is therefore recognized as an important therapeutic target tissue for insulin resistance [11,44]. GLUT4 plays a crucial role in maintaining systemic glucose homeostasis. It is mostly intracellular in the unstimulated state but is acutely redistributed to the PM in response to insulin, contraction, and other stimuli [9]. The amount of glucose uptake is determined by the number of GLUT4 molecules on the muscle cell membranes [36,45]. Relevant studies reported that GLUT4 translocation and fusion with PM were the main rate-limiting steps for glucose disposal [46,47]. The existing synthetic antidiabetic drugs often exhibit side effects or resistance that pose a huge challenge to managing diabetes [43]. It is, therefore, necessary to find better medicines from herbs or natural products. Consequently, we extracted the effective products from the natural medicinal plant Folium Sennae. We investigated the potential activity of FSE on glucose uptake in L6 rat skeletal muscle cells in regard to GLUT4 expression, translocation, and fusion with PM and the participation of Ca^2+^ in this process.

It has been reported that an ethanolic extract of the leaves of *Senna surattensis* increased glucose uptake in an isolated rat hemidiaphragm model [34]. However, little is known about how FSE exerts its hypoglycemic mechanism in vivo. In this study, we first investigated the effect of FSE on the glucose uptake of L6 cells. Consistent with the reported results, FSE significantly increased glucose uptake in L6 cells. We also discovered that FSE upregulated GLUT4 mRNA and protein expression in GLUT4 molecular assays. The results suggested that there was some connection between FSE-induced glucose uptake and intracellular GLUT4 and IRAP motion (Figure 1).

Next, in order to observe the translocation of GLUT4 caused by FSE, we studied L6-IRAP-mOrange cells under confocal microscopy. Due to the high colocalization of intracellular IRAP and GLUT4 [48], IRAP was used as a reporter molecule to track and quantify dynamic information about GLUT4 in real time. Our results showed that FSE promoted the increase in IRAP expression in cytosol. By collecting red fluorescence in the periphery of the cells, we found marked increases in IRAP in the PM region, which indicated that FSE promoted GLUT4 translocation (Figure 2A,B). In this way, FSE-mediated increases in GLUT4 transcription and translation are able to provide more GLUT4 traffic to the PM, triggering glucose uptake in L6 cells. Previous studies have shown that increasing intracellular cytosolic Ca^2+^ concentration increases cell surface GLUT4 levels [19]. We observed that upon intracellular Ca^2+^ loading, the fluorescent indicator also increased almost synchronously with GLUT4. This phenomenon implied that FSE-enhanced GLUT4 translocation may mobilize intracellular Ca^2+^.

We then sought to search for the signal transduction pathways by which FSE stimulates GLUT4 expression and translocation. There are various signaling pathways which are involved in GLUT4 translocation and/or expression, such as the AMPK pathway, the PI3K/Akt pathway, and the PKC pathway. AMPK, a key signaling molecule stimulated by muscle contraction, activates to cause GLUT4 translocation [45]. GLUT4 is a member of the glycolytic enzyme genes. Its expression was activated under the effect of the AMPK agonist AICAR [49]. PI3K can catalyze the phosphorylation of phosphatidylinositol 4,5-diphosphate (PIP2) to PIP3, activating the downstream signaling factor Akt by phosphorylation, thereby promoting GLUT4 translocation to the PM to absorb glucose into the muscle [44,50]. The PKCα inhibitor dioleoyl phosphoethanolamine retained cell surface GLUT4 by inhibiting PKCα-driven internalization in adipocytes. PKC β and λ were involved in the insulin signaling cascade causing PKC-meditated GLUT4 traffic in skeletal muscle cells [19,51]. When we studied which of the three signaling pathways was/were involved in FSE-mediated glucose uptake, we found that both Compound C and Gö6983 remarkably inhibited the promotion by FSE of GLUT4 translocation and expression, but the inhibition effect of Wortmannin was relatively weak (Figure 4A,B). This suggested that the PI3K/Akt pathway was not dominant in the process of FSE-mediated GLUT4 translocation. Furthermore, the observation that FSE increased the phosphorylation of AMPK, Akt, and PKC proteins in L6 cells further conjectured that FSE-induced glucose uptake was associated with the AMPK, Akt, and PKC signaling pathways (Figure 4C–E).

To study the relationship between FSE-mediated GLUT4 translocation and Ca^2+^, we partially removed Ca^2+^ and found that 0 mM extracellular Ca^2+^ partially inhibited FSE-induced Ca^2+^ elevation but did not affect GLUT4 translocation (Figure 3A). While the intracellular and extracellular Ca^2+^ were all chelated, the FSE-induced Ca^2+^ increase was almost completely blocked and the GLUT4 translocation was also severely inhibited (Figure 3B), and it was certified that cytosolic Ca^2+^ participated in FSE-induced GLUT4 translocation. In order to examine the source of cytosolic Ca^2+^, internal Ca^2+^ release was also studied, in addition to external Ca^2+^ influx. IP3R and RyR are two important barriers to the release of Ca^2+^ from intracellular Ca^2+^ stores, controlling the output of internal Ca^2+^ [18]. Similar to 0 mM Ca^2+^, FSE-mediated Ca^2+^ increase was obviously inhibited after 2-APB blocking IP3R. Nevertheless, GLUT4 translocation was not altered (Figure 6A). However, the inhibition of RyR did not have any impact on FSE-triggered Ca^2+^ elevation or GLUT4 translocation. These results showed that FSE-induced Ca^2+^ elevation may be related to IP3R (Figure 6B). To explain why simply blocking the external or internal Ca^2+^ source to reduce cytosolic Ca^2+^ concentration does not inhibit GLUT4 translocation of FSE, we propose that the lower Ca^2+^ concentration can cause the GLUT4 translocation process via FSE while Ca^2+^ can be obtained from another source, even if one of the sources was blocked.

G protein and PLC are upstream of the PKC signaling pathway and IP3R [52]. This provides a potential breakthrough point for simultaneously refining the pathway of FSE-regulated intracellular Ca^2+^ release and GLUT4 translocation. We found that G protein and PLC inhibitors disrupted the FSE-induced Ca^2+^ increase and displayed varying degrees of suppression efficacy. This was demonstrated by the observation that FSE induced intracellular Ca^2+^ release through the G protein-PLC-IP3-IP3R pathway. Regarding the partial inhibition of G protein and PLC inhibitors of GLUT4 translocation, we concluded that FSE-regulated GLUT4 translocation not only depends on one pathway, but may involve two or more signaling pathways (Figure 5A–C).

In addition, our studies testified that FSE increases Ca^2+^ to enhance GLUT4 insertion into the PM and increases glucose uptake. GLUT4 fusion is the last step in glucose uptake and several studies have reported a key role for Ca^2+^/calmodulin in the late stages of GLUT4 vesicle docking/fusion [53,54]. As shown in Figure 7 and Figure 8, both FSE-mediated GLUT4-PM fusion and glucose uptake were somewhat attenuated under conditions of abolishment of extracellular Ca^2+^. When free Ca^2+^ ions were fully chelated by EGTA and BAPTA-AM, FSE-induced membrane fusion and especially glucose uptake were also seriously diminished. It can be seen that FSE-mediated membrane fusion and glucose uptake require Ca^2+^, which makes Ca^2+^ indispensable in these steps.

Throughout the course of this research, the signaling pathways induced by FSE-mediated glucose uptake were found to be quite diverse. This diversity may be attributed to the differences in the active ingredients of FSE. Based on the components of FSE as described above, many interesting results have been found. For example, through inhibiting mitochondrial complex I activity, emodin increases cellular ROS and Ca^2+^ influx to activate AMPK, causing GLUT4 translocation and glucose uptake [55]. Emodin significantly improved the blood glucose of diabetic rats by activating the PI3K/Akt/GSK-3β signaling pathway [56]. Isolated chrysophanol from rhubarb rhizome increased tyrosine phosphorylation of the insulin receptor (IR) and accelerated GLUT4 mRNA expression [57]. Aloe-emodin glycosides stimulated glucose transport and glycogen storage through PI3K-dependent mechanisms in L6 myotubes [58]. Long-term dietary supplementation of kaempferol promoted the expression of AMPK and GLUT4 in skeletal muscles, thereby preventing hyperglycemia in middle-aged obese mice [59]. These statements implied that the FSE-regulated glucose pathway is a complex and vast network. Our study shows for the first time that FSE regulates glucose metabolism through the PKC pathway, but it is not clear which active ingredient plays a role in it and is worth further exploration. Besides, this research on FSE is limited to the cellular level; hence the hypoglycemic effect at the animal level remains to be explored.

**Figure 9:**
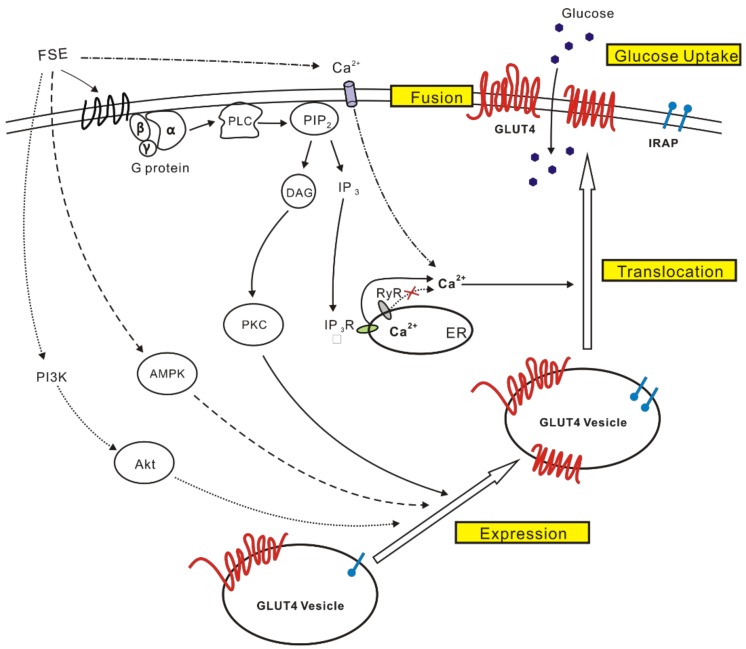
Proposed mechanism of FSE for the enhancement of glucose uptake in L6 cells. FSE promotes GLUT4 expression and translocation via the activation the AMPK, IP3K/Akt, and G protein-PLC-PKC pathways. Moreover, the increase in cytosolic Ca^2+^ induced by the G-protein-PLC-IP3-IP3R-Ca^2+^ pathway or/and by extracellular Ca^2+^ influx assists GLUT4 trafficking and fusion to PM, ultimately triggering glucose uptake.

In conclusion, FSE promoted GLUT4 expression and translocation through the AMPK, Akt, and G protein-PLC-PKC pathways. FSE increased Ca^2+^ concentration by external Ca^2+^ influx and internal Ca^2+^ release of G protein-PLC-IP3-IP3R signals to assist GLUT4 traffic and PM fusion, ultimately leading to glucose uptake (Figure 9). Therefore, FSE is expected to become an effective drug for the treatment of insulin resistance.

## 4. Materials and methods

### 4.1. Reagents and Solutions

Alpha-MEM (α-MEM) and penicillin streptomycin solution were purchased from Gibco (Gran Island, NY, USA). Fetal bovine serum (FBS) was purchased from Hyclone (Logan, UT, USA). The glucose assay kit was purchased from Cayman Chemical Company (Ann Arbor, MI, USA). Fluo-4 AM was obtained from Invitrogen (Camarillo, CA, USA). Compound C was purchased from Selleckchem (Houston, TX, USA). Wortmannin, BAPTA-AM, U73122, Ryanodine, and 2-APB were purchased from Sigma (St. Louis, MO, USA). Gallein and PTX were purchased from Tocris Bioscience (Bristol, UK). Gö6983 was from EMD Millipore (Billerica, MA, USA). GLUT4 antibody, β-actin antibody, phospho-AMPKα (Thr172) antibody, AMPK antibody, phospho-Akt (Ser473) antibody, Anti-Akt antibody, and phospho-PKC pan (Thr410) antibody were from Cell Signaling Technology (Beverly, MA, USA). HRP goat anti-mouse and goat anti-rabbit IgG antibody were from CWBIO (Beijing, China), anti-c-myc mouse monoclonal antibody and FITC antibody were purchased from TransGen Biotech (Beijing, China), Phorbol 12-myristate 13-acetate (PMA) was purchased from Ascent Scientific (Cambridge, MA), metformin and insulin were obtained from Yuanye biological (shanghai, China). 2 mM Ca^2+^ (pss): 135 NaCl, 5 KCl, 1 MgCl_2_, 2 CaCl_2_, 10 HEPES, and 10 glucose (pH 7.4). 0 mM Ca^2+^-EGTA: 135 NaCl, 5 KCl, 1 MgCl_2_, 0.5 EGTA, 10 HEPES, and 10 glucose (pH 7.4). PBS buffer: 137 NaCl, 2.7 KCl, 10 Na_2_HPO_4_, 2 KH_2_PO_4_, and the volume is adjusted to 1 L with ultrapure water (PH 7.4).

### 4.2. Plant Collection and Preparation

Dried plant material was ground into a fine powder using a commercial electric blender and stored in air-tight containers. Fifty grams of leaf material was soaked in 500 mL of 70% industrial ethanol for 30 h, and then refluxed in a 60 °C water bath. The extraction procedure was repeated three times in order to extract as much of the components as possible. The extract was filtered using Whatman no. 1 filter paper and dried under a stream of cold air. The ethanolic extract of Folium Sennae (FSE) was dissolved in 3% dimethylsulfoxide (DMSO) for biological detection.

### 4.3. L6 Cell Culture and Treatment

L6 myoblasts were grown in α-MEM containing 10% (*v*/*v*) FBS, 100 units/mL penicillin, and 100 μg/mL streptomycin in 5% CO_2_ at 37 °C. L6 cells were harvested after about 80% confluent and used in all experiments. Prior to any treatment, the cells were serum-deprived for 2 h. After treatment, the cells were divided into different drug treatment groups.

### 4.4. Amount of Glucose Uptake into Cells

The glucose uptake stimulatory effect of FSE was determined in L6 cells by a previously reported method [60] with slight modifications [61,62]. Briefly, L6 cells were grown in 96-well culture plates at 37 °C under 5% CO_2_ and cultured for 3–4 days until the cells were confluent. Next, cells were starved for 2 h with 100 μL serum-free α-MEM medium and then treated with 60 μg/mL FSE or 100 nM insulin or vehicle control dissolved in 100 μL serum-free α-MEM medium for 1 h. Insulin was used as positive control. The concentration of glucose remaining in the media was determined according to the glucose assay kit manufacturer’s instructions using an Infinite M200 Pro microplate reader (Tecan, Croedig, Austria) with a 505 nm wavelength. Meanwhile, cell numbers from each group were analyzed using tetrazolium salt (3-(4,5-dimethylthiazol-2-yl)-2,5-diphenyltetrazolium) bromide (MTT). For analysis, 20 µL of MTT substrate was added to each well and the plates were incubated for an additional 4 h at 37 °C with 5% CO_2_. The medium was removed, and the cells were solubilized in 150 µL DMSO. The colorimetric analysis was performed at a wavelength of 492 nm. The amount of glucose uptake in each group was calculated by using the amount of glucose uptake for the corresponding cell counts. Three independent experiments were conducted, comparing the control group (control), the insulin group (insulin), and the added drug group (FSE).

### 4.5. Western Blotting Analyses

L6 cells were serum-deprived for 2 h, then treated with FSE (60 μg/mL for 30 min), insulin (100 nM for 30 min), PMA (200 nM for 4 h) or metformin (100 μg/mL overnight), Wortmannin (100 nM for 30 min), Gö6983 (10 μM for 30 min), Compound C (10 μM for 30 min). Next, the cells were placed on ice, washed three times with cold PBS, and treated with a protease inhibitor cocktail (Roche, Basel, Switzerland) and phosphatase inhibitor cocktail (Selleckchem, Houston, TX, USA) at 4 °C. Cells were then lysed as described previously [60]. After cell debris was removed, supernatants containing proteins were collected. Equal amounts of protein were separated by 10% (*v*/*v*) SDS-PAGE and transferred to polyvinylidene difluoride (PVDF) membranes. Blots were incubated with 5% BSA blocking solution at 4 °C, overnight with a primary antibody (1:1000), and then incubated with corresponding horseradish peroxidase-conjugated secondary antibody (1:10,000) for 1 h at room temperature. The intensity of protein bands was quantitated using a ChemiDoc XRS system (Bio-Rad, Hercules, CA, USA).

### 4.6. RT-PCR

The L6 cells were subjected to the same western blotting operation described above and divided into control, insulin, and FSE groups. Total RNA was extracted using TRIzol reagent (Invitrogen) including chloroform extraction and isopropanol precipitation. The RNA initial extract of each sample was washed and dried with 75% ethanol and then dissolved in 50 μL DEPC water. The quality of each RNA extraction was confirmed by electrophoresis on a 1% agarose gel. Next, 2 μg of total RNA from each sample was reverse-transcribed using the RevertAid First Strand cDNA Synthesis Kit (Thermo Scientific, Wilmington, DE, USA) in a 20 μL reaction according to the manufacturer’s protocol. The PCR conditions were set as follows: initial activation of Taq polymerase at 95 °C for 10 min, 35 cycles of PCR amplification at 95 °C for 15 s, and annealing/elongation at 60 °C for 1 min. cDNA products were diluted with RNAse-free water, and then real-time PCR was performed using the FastStart Universal SYBR Green PCR Master ROX (Roche) system on the 7500 Fast Real-Time PCR System instrument (Applied Biosystems, Foster City, CA, USA). Primer sequences were as follows: rat GAPDH (NCBI RefSeq NM_017008.4), F: 5′-TACAGCAACAGGGTGGTGGAC-3′, R: 5′-GGGATGGAATTGTGAGGGAGA-3′; rat GLUT4 (NCBI RefSeq NM_012751.1), F: 5′-CTTCCTTCTATTTGCCGTCCTC-3′, R: 5′-GCTGCTGTTTCCTTCATCCTG-3′; rat IRAP (NCBI Refseq NM_001113403.2), F: 5′-GTGGGGACTAAGGGCGAAAA-3′ R:5′-CATACATCCGGACCTCCACG-3′. Relative quantification results obtained for the target genes were normalized using the 2^-ΔΔCT^ method. 

### 4.7. Fluorescence Microscopy for Detection of IRAP Translocation and Ca^2+^

L6 cells were transfected with pIRAP-mOrange cDNAs (presented by Professor Xu Tao, Chinese Academy of Sciences) using Lipofectamine 2000 as per the manufacturer’s protocol [2]. Stably expressing IRAP-mOrange of L6 cells (L6 IRAP-mOrange) were seeded into a glass slide and incubated overnight until differentiated and confluent over the slides. Cells were starved in serum-free α-MEM for 2 h. For loading the dye, cells were incubated in 2 μM fluo4-AM (Invitrogen, Carlsbad, CA, USA) for 20 min at 37 °C, followed by a wash with PSS, and then treated with 60 μg/mL FSE or other related signaling pathway protein inhibitors. Red fluorescence (555 nm) and green fluorescence (488 nm) images of cells were simultaneously taken with the LSM700 laser scanning confocal microscope (Carl Zeiss, Jena, Germany). The IRAP-mOrange translocation and intracellular Ca^2+^ levels were monitored as spatial and temporal changes in the fluorescence intensity of the indicator mOrange and dye Fluo4, respectively. In the absence of stimulation, GLUT4 and IRAP are present in intracellular GLUT storage vesicles (GSVs). Many studies have reported that GLUT4 and IRAP displayed a high co-localization relationship [63], thus, detecting the IRAP can indirectly reflect the localization of GLUT4. The images were captured with 555 nm or 488 nm excitation laser every 10 s in the first 5 min and then every 5 min over 25 min.

### 4.8. Immunofluorescence of GLUT4 Fusion with PM

L6 cells were transfected by lentivirus with GV348 plasmid DNA encoding myc, GLUT4, and mOrange constructs of the myc-GLUT4-mOrange fusion protein. GLUT4myc cDNA was constructed by inserting the human c-myc epitope (14 amino acids) into the first ectodomain of GLUT4, as described [64]. Live cell imaging was performed to measure membrane fusion and GLUT4 trafficking with myc and mOrange as molecular probes, respectively [42]. A single clone containing the highest fluorescence intensity was selected and used for the following experiments. The GV348-myc-GLUT4-mOrange of L6 cells were seeded onto the glass slide, incubated until they reached 80% confluence, and then serum-deprived for 2 h. In addition to the control group and the 100 nM insulin-positive control group of 2 mM Ca^2+^, the cells were treated with 60 μg/mL FSE under the conditions of 2 mM Ca^2+^, 0 mM Ca^2+^, and 0 mM Ca^2+^+BAPTA-AM, respectively. After 30 min of stimulation, the cells were fixed with 4% paraformaldehyde. Then, 50 mM glycine was used to remove impurities which would cause background signals. Then samples were blocked with PBS containing with 2% FBS for 1 h. Afterward, cells were labeled with anti-c-myc mouse monoclonal antibody (1:200) and goat anti-mouse IgG (H+L) FITC-conjugated secondary antibody (1:200). Cell images of green fluorescent protein (FITC) and mOrange fluorescence were measured by the LSM700 microscope (Carl Zeiss).

### 4.9. Statistical Analysis

Data are presented as the mean  ±  SEM of at least three independent experiments. All statistical analyses were performed using OriginPro 8 (OriginLab, Northampton, MA, USA). Multigroup comparisons were conducted by one-way analysis of variance and differences between two group means were compared by Student’s *t*-test. *p* values < 0.05 were considered to represent statistical significance. The n values represent the number of cells.

## 5. Conclusions

The present study showed that FSE promoted GLUT4 expression and translocation in L6 cells via the AMPK, PI3K/Akt, and G protein-PLC-PKC signaling pathways. It also increased Ca^2+^ concentration in the form of external Ca^2+^ influx and Ca^2+^ pool release, assisting GLUT4-PM fusion to enhance glucose uptake. These results suggested the possibility of FSE being used as a novel hypoglycemic agent for the treatment of T2DM.

## Supplementary Materials

Supplementary materials are available online. Figure S1: MTT test with different treatment groups. Figure S2: Comparative images with insulin treatment.
