# Supplementary material for: Ethanolic Extract of Folium Sennae Mediates the Glucose Uptake of L6 Cells by GLUT4 and Ca2+
Source: Molecules. 2018 Nov 9;23(11):2934. doi: 10.3390/molecules23112934 (PMC6278344; doi:10.3390/molecules23112934)
Supplement: Supplementary file 1 [file molecules-23-02934-s001.zip › Ethanolic Extract of Folium Sennae Mediates the Glucose Uptake of L6 Cells by GLUT4 and Ca2+Supplementary material-Edited.pdf]

Supporting Information

Ethanolic Extract of Folium Sennae Mediates the Glucose Uptake of L6 Cells by GLUT4 and Ca^{2+}

Ping Zhao [1,3,*], Qian Ming [1], Junying Qiu [1], Di Tian [1], Jia Liu [1], Jinhua Shen [1], Qing-Hua Liu [1], Xinzhou Yang [2,3,*]

[1] Institute for Medical Biology & Hubei Provincial Key Laboratory for Protection and Application of Special Plants in the Wuling Area of China, College of Life Sciences, South-Central University for Nationalities, Wuhan 430074, China

[2] School of Pharmaceutical Sciences, South-Central University for Nationalities, 182 Min-Zu Road, Wuhan 430074, China

[3] National Demonstration Center for Experimental Ethnopharmacology Education, South-Central University for Nationalities, Wuhan 430074, China

* Correspondence: p.zhao@scuec.edu.cn (P.Z); xzyang@scuec.edu.cn (X.Z.Y)

*Corresponding authors at: School of Life Sciences, South-Central University for Nationalities, 182 Minzu Road, Wuhan 430074, China, Tel.: +86 27 67842576, Fax: +86 27 67841906; School of Pharmaceutical Sciences, South-Central University for Nationalities, 182 Minzu Road, Wuhan 430074, China, Tel.: +8627 67841196, Fax: +86 27 67841196.

**Both authors contributed equally to this paper. Affiliations of the authors are in no particular order.

Supporting Information Contents

S1. MTT test with different treatment groups

S2. Comparative images with insulin treatment

S1. MTT test with different treatment groups

L6 cells were grown in 96-well culture plates at 37 ℃ under 5% CO2 and cultured for 3-4 days until the cells were confluent. Next, cells were starved for 2 hours with 100 μL serum-free α-MEM medium and then treated with 30, 60 and 120 μg/mL FSE or 100 nM insulin or vehicle control dissolved in 100 μL serum-free α-MEM medium for 1 hour. Insulin was used as positive control. The concentration of glucose remaining in the media was determined according to the glucose assay kit manufacturer's instructions using an Infinite M200 Pro microplate reader (Tecan, Croedig, Austria) with a 505 nm wavelength. Meanwhile, cell numbers from each group were analyzed using tetrazolium salt (3-(4,5-dimethylthiazol-2-yl)-2,5-diphenyltetrazolium) bromide (MTT). For analysis, 20 μL of MTT substrate was added to each well and the plates were incubated for an additional 4 hours at 37℃ with 5% CO2. The medium was removed, and the cells were solubilized in 150 μL DMSO. The colorimetric analysis was performed at a wavelength of 492 nm. The amount of glucose uptake in each group was calculated by using the amount of glucose uptake for the corresponding cell counts. Three independent experiments were conducted, comparing the control group (control), the insulin group (insulin), and the added drug group (FSE).

From the results of Figure S1, we observed that 100 nM insulin and different concentrations of FSE were not cytotoxic to L6 cells. And it is interesting that the number of cells increased slightly after 100 nM insulin incubation.

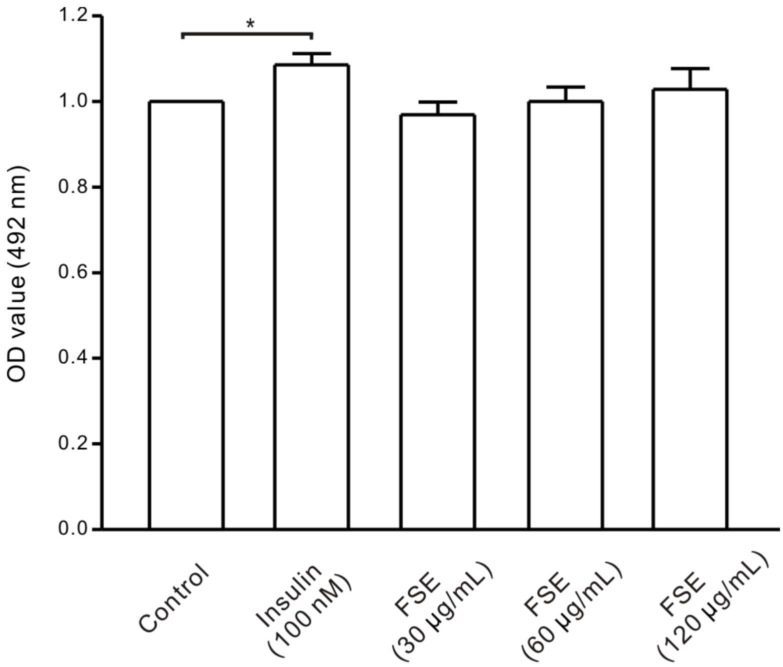

S1. Toxicity of drugs to L6 cells by MTT assay. L6 cells were starved for 2 hours, and then treated with 30, 60 and 120 μg/mL FSE or 100 nM insulin for 30 minutes, the cells were incubated with MTT for an additional 4 hours, the colorimetric analysis was performed at a wavelength of 492 nm. Datas were obtained from three independent repeated experiments. Significance analysis: $*P < 0.05$.

S2. Comparative images with insulin treatment

L6 cells were transfected with pIRAP-mOrange cDNAs (presented by Professor Xu Tao, Chinese Academy of Sciences) using Lipofectamine 2000 as per the manufacturer's protocol. Stably expressing IRAP-mOrange of L6 cells (L6 IRAP-mOrange) were seeded into a glass slide and incubated overnight until differentiated and confluent over the slides. Cells were starved in serum-free α-MEM for 2 hours. For loading the dye, cells were incubated in 2 μM fluo4-AM (Invitrogen, Carlsbad, CA, USA) for 20 minutes at 37 ℃, followed by a wash with PSS, and then treated with 100 nM insulin. Red fluorescence (555 nm) and green fluorescence (488 nm) images of cells were taken with the LSM700 laser scanning confocal microscope (Carl Zeiss, Jena, Germany), respectively. The IRAP-mOrange translocation and intracellular Ca^{2+} levels were monitored as spatial and temporal changes in the fluorescence intensity of the indicator mOrange and dye Fluo4, respectively.

The IRAP fluorescence intensity in cytoplasm was obviously raised after the addition of 100 nM insulin and a substantial amount of red fluorescence accumulated at the cell periphery as revealed by IRAP-mOrange signals. In addition, we observed that the green fluorescence of Ca^{2+} was densely distributed in the cells. Our studies suggested that 100 nM insulin not only stimulating GLUT4 translocation but also increasing intracellular Ca^{2+} levels.

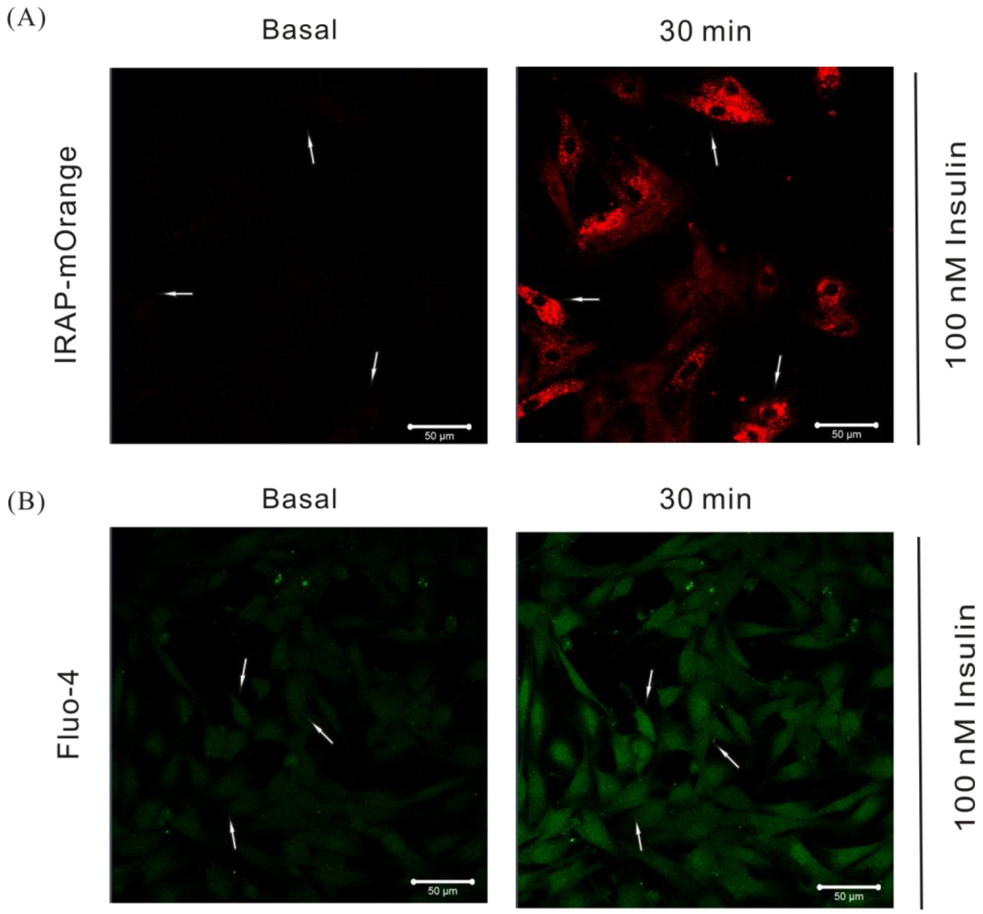

S2. Stimulating effects of insulin on GLUT4 translocation and intracellular Ca^{2+} level. (A)The red fluorescence of IRAP-mOrange stably expressed in L6 cells and the green fluorescence of Ca^{2+} were observed by confocal microscope, respectively. Scale bar = 50 µm. (B) Intracellular Ca^{2+} was stained with Flou-4 AM for 20 minutes, followed by stimulation with 100 nM for 30 minutes. IRAP-mOrange fluorescence intensity and intracellular Ca^{2+} fluorescence concentration were detected at excitation wavelengths of 555 nm and 488 nm, respectively.
